# ‘Locked rehabilitation’: a need for clarification

**DOI:** 10.1192/pb.bp.114.049726

**Published:** 2016-02

**Authors:** Stephen Dye, Lucy Smyth, Stephen Pereira

**Affiliations:** 1 Norfolk and Suffolk NHS Foundation Trust, Ipswich; 2 University College London; 3 Keats House, London

## Abstract

Recently, the term ‘locked rehabilitation’ has spread from commissioning to now also clinical parlance. This is without any clear service description or category of patient which this service manages. Differences between this new term and an established definition of low secure services are examined and reasons for the introduction of this terminology are discussed. This is contextualised within service development, payment by results and measures of quality. It is argued that there is a need for ongoing measurements of types of patients admitted to, and treatments offered by, this ‘new’ ward, as well as those within psychiatric intensive and low secure care services.

Since Dominic Beer's thoughtful editorial in *BJPsych Bulletin* outlining the state of psychiatric intensive care and low secure services in the UK,^1^ a significant change has occurred. As described by Dix^2^ and Chukwuma,^3^ over the past few years a new service type of ‘locked rehabilitation’ has emerged within the UK. However, services that these units provide have not yet been defined. Whereas they are ‘standalone units at the heart of communities’,^2^ much of their clinical focus overlaps with that of low secure units (LSUs). Chukwuma^3^ suggests that although ‘locked rehabilitation units’ and LSUs differ slightly in areas of physical security, locked rehabilitation units tend to follow guidance set out by the Department of Health in the national minimum standards (NMS) for low secure services.^4^ Furthermore, it is suggested that ‘locked rehabilitation units’ also share the treatment objectives and provide similar treatment and care to LSUs, in that they aim to provide long-term, multidisciplinary, recovery-oriented treatment and rehabilitation.

## ‘Locked rehabilitation’ and low secure units: do they differ?

So what are the differences between the two services? Is locked rehabilitation the new low secure care? Are LSUs really forensic locked rehabilitation units? Do they both treat similar types of patient and fulfil the same role in different guises? Essentially, these questions emanate from a single question: on what basis was this ‘new’ service named and developed?

### Service development

In answering this question it may be useful to examine some of the historical development of low secure care as well as psychiatric intensive care units (PICUs) in the UK. The impetus for centrally funded medium secure forensic services came from the Butler report.^5^ Although this spurred development of regional secure units, it did not take into account the provision of care for general psychiatric in-patients who needed secure care for short periods of time before returning to an open setting. This was addressed by the Reed report when the concept of what we recognise as psychiatric intensive care was first acknowledged.^6^ The Reed report also stated that ‘although more medium secure provision is required, there also needs to be a range of facilities between this and open local settings in order to meet the needs of the population’. This gave rise to what came to be defined as low secure provision within national standards.^4^

Despite definitions of PICUs and LSUs laid out in the Department of Health guidance,^4^ ambiguity remained concerning the role of low secure services. A report from the Centre for Mental Health in 2011 stated that ‘low secure is more of a concept than a title for a discrete type of provision’.^7^ It recommended reviewing the roles of low secure and step-down care to inform commissioning decisions and systems. A big difference between the development of regional medium secure services and that of local PICUs and LSUs was that although the former were centrally funded, it was up to each local area to respond to the Reed report (and associated cost implications) how it saw fit. This led to a variety of service types arising that were not appropriately defined with adequate criteria. This assortment of services aimed to meet the needs of patients and provide the full ‘range of facilities’ demanded by the Butler report. Thus, each area defined low secure care differently and attempted to provide a different service that was believed to meet the needs of patients within their region. Perhaps, over time, these services progressed to define the types of patients treated within them, rather than meeting any changing needs of the local population. Non-statutory providers also recognised an unmet service need and responded with services that would meet this need. Thus, it is not surprising that confusion arose: from different types of patients in different but appropriate kinds of services, different types of patients in inappropriate services, the same type of patient being managed in different modes of service, or even the same form of service managing different patient types! So a variety of estates exist within ‘low secure’ that attempt to cover all bases with regard to differing needs, but with little clarity, let alone specificity.

### Funding as the source of new labelling?

Yet again, history seems to be repeating itself: a key difference between LSUs and locked rehabilitation relates to how these services are funded. Whereas LSUs are currently commissioned more centrally by NHS England as part of the forensic pathway, locked rehabilitation units appear to be locally commissioned as part of the acute care pathway.^3^ Judging by its title, *National Minimum Standards for General Adult Services in Psychiatric Intensive Care Units (PICU) and Low Secure Environments*,^4^ the clinical guidance published in 2002 was aimed at locally commissioned general adult services. So, locked rehabilitation may be what general adult psychiatrists have always known and recognised as low secure care. It is possible that this service split has only developed following funding centralisation and ‘forensification’ of some established units which have taken on the mantle of more ‘modern’ low secure care. It may be that commissioning arrangements will be reviewed in the near future and funding streams become clearer. Perhaps it is time for a clinical redefinition of low secure care as, by proactively addressing sociopolitical challenges and changes within the UK health service, it may actually be that some previously established LSUs have developed unknowingly as the ‘new kids on the block’ (to quote Dix^2^) and that locked rehabilitation is meeting some of the service and patient needs following these changes.

## Endorsing change – and learning from it

If history is repeating itself, what can be learnt from it and do we need to modify our approach to changing services? A major change in funding of mental health services is the introduction of a payment by results (PbR) system that is based on clusters of patients progressing along pathways that are set by commissioners and providers. These patient clusters are supposedly derived from patient need as opposed to diagnosis and are created by a clustering tool based on the Health of the Nation Outcome Scales (HoNOS).^8^ Difficulties have been outlined with this approach and an alternative has been suggested within a PICU environment – identifying patient needs' types and using lean management techniques to refine pathways.^9^ This approach can also be applied to patients within low security/locked rehabilitation. The recently updated minimum standards for psychiatric intensive care has proposed that there are three types of patient within low security: those who are descending mental health security, those who are transferred from the criminal justice service (for lower-level offences) and those who present significant challenge to other in-patient settings with a risk profile similar to the other two groups.^10^ The commonality for all three groups is that the active risk assessment on admission is no more than potential for actual bodily harm or similar offences.

Despite this commonality, the needs of these types of patient are different and pathways they follow may be better described and defined by patient need-typing as opposed to PbR-clustering. Provision of care packages based on commonalities of clusters for individuals who have varied pathways through services (dependent on need for different care) is a convoluted and imprecise manner of meeting care needs. Although there may be commonality in the active risk on admission, this may be managed in differing ways for different types of patients dependent on their varying needs. Identification and use of patient types and development of care packages could well give a clue as to the distinction between locked rehabilitation and low secure services and how they could meet needs of particular patient types. Perhaps the ‘modern’ low secure care is associated with a different patient type than the as yet undefined locked rehabilitation? This may also support Dix's prediction that differences in cost between LSUs and locked rehabilitation units may account for changes in LSU provision (an average night's accommodation and treatment in an LSU costs £500, compared with £300 for accommodation and treatment in a locked rehabilitation unit).^2^

### Finally, evidence-based care

Terminology is interesting but how does that help our patients? To answer this we need evidence. Historically, there have been two major national surveys of PICUs/LSUs. The first, by Beer *et al*,^11^ identified 110 PICUs in the UK. The results of this survey showed that PICUs varied in many aspects of structure and function, including size, level of security, admission of informal patients and length of patient stay. Concerns regarding the lack of local and national guidance relating to operational definitions of PICUs, low secure provision, policies and guidelines were highlighted.^12^ The National Service Framework also recognised the need for psychiatric intensive and low secure care.^13^ Publication of national minimum standards that separated PICUs and LSUs followed, giving some clinical operational guidance which has recently been updated from a PICU perspective.^4,10^

Shortly after the publication of the initial standards, the most comprehensive national survey of PICUs and LSUs in the UK was carried out.^14,15^ This survey resulted in the development of a national data-set for PICUs and LSUs, together with a more comprehensive understanding of the different service provision and patient characteristics within these units. A total of 307 units in the UK were identified, 170 PICUs and 137 LSUs. The results of this survey indicated that there were significant differences between PICUs and LSUs and between patients treated in these units. It also identified ongoing inconsistency in areas of operation and structure: PICUs offered a more time-limited, medically oriented treatment, with a higher number of qualified staff than LSUs. The average length of stay on a PICU was found to be 27 days and over 90% of service provision was National Health Service (NHS)-funded. The research was consistent with previous studies of PICUs, which highlighted their role in offering time-limited care for patients experiencing a severe and acute episode of mental illness. LSUs were found to place more emphasis on long-term therapeutic treatment and rehabilitation, and to provide a step down from higher levels of security. This was illustrated by the longer length of stay (on average 358 days), higher levels of psychology, social work and occupational therapy input and fewer medical interventions. The survey identified a link between LSUs and the independent sector as there was more non-NHS service provision for LSUs than PICUs (28% *v.* 10%).

These surveys contributed to and highlighted separation of PICUs and LSUs based on patient need. At present it appears that, perhaps driven to an extent by funding arrangements, there is confusion in definitions of low secure care. There has been development of a newly named service without evidence that it meets a different patient need. Perhaps by examining patient types, it may well be discovered that specific types of patient need are treated or should be treated within specific different services.

## A way forward

The landscape of service provision within PICU and LSU has certainly changed since the first publication of the NMS. Given the introduction of a supposedly different service that has yet to be appropriately defined, there is a need for further clarification of what is available for different types of patient within this resource. Not only this, but standards and form of service require assessment both for uniformity and quality.

The Royal College of Psychiatrists' accreditation scheme (AIMS) in collaboration with the National Association of Psychiatric Intensive Care and Low Secure Units (NAPICU) has developed AIMS-PICU.^16^ This is a standards-based accreditation programme designed to improve the quality of care in in-patient mental health wards. It has gone some way to establishing measures of ‘quality’ within a PICU but not on a national basis as data are owned by individual participating units. It also uses service and environmental quality measures as opposed to examining types of patient need and how this is met. The development of these standards has been possible because of the robust definition of psychiatric intensive care. This in itself was led by clarity of type of service need for defined varieties of patient need which was discovered through research. Without evidence and specificity of patient need, services cannot be clearly defined or measured. Although an attempt has been made to identify need using the PbR framework, this feels more like the tail wagging the dog. Given the current lack of definition for a ‘new’ type of service, the time has surely come for research to be re-visited.

Development of a framework of guidance and quality standards for secure services and specific care packages with specified outcomes for care has been recommended in the past.^7^ In our opinion, following the publication of revised PICU standards, a specific rolling arrangement of measurements of clinical provision for different types of patients within PICU, LSU and locked rehabilitation is now desperately needed. This will allow for benchmarking both locally and nationally. If this is adopted, initially the definition of locked rehabilitation and the type of patients served may become clearer. As a rolling venture that is repeated at regular intervals (e.g. every 12–18 months), this would also go some way to identifying and predicting unmet need and thus to developing more appropriate services. In this way, as well as providing some clarification, any further changes may be founded on clinical evidence as opposed to being politically or financially motivated. Thus, as well as clarity, explicit types of patient need will define specificity of services and then quality can be developed and measured appropriately.
